# Food handling practice and associated factors among food handlers in public food establishments, Northwest Ethiopia

**DOI:** 10.1186/s13104-019-4047-0

**Published:** 2019-01-14

**Authors:** Fasikaw Adbarie Chekol, Melkitu Fentie Melak, Aysheshim Kassahun Belew, Ejigu Gebeye Zeleke

**Affiliations:** 1 Dabat District Health Office, North Gondar Zonal Health Office, Amhara Regional Health Bureau, Dabat, Ethiopia; 2 Institute of Public Health Department of Human Nutrition, College of Medicine and Health Sciences, University of Gondar, Gondar, Ethiopia; 3 Institute of Public Health Department of Epidemiology and Biostatistics, College of Medicine and Health Sciences, University of Gondar, Gondar, Ethiopia

**Keywords:** Food handling practice, Food handler, Debark, Ethiopia

## Abstract

**Objective:**

The objective of this study was to assess food handling practice and associated factors among food handlers in public food establishments, Northwest Ethiopia.

**Results:**

In this study a total of 416 food handlers were participated with a response rate of 416 (98.6%). Proportion of good food handling practice was 167 (40.1%) [95% CI (confidence interval): 35.10, 44.50]. Work experience [AOR (adjusted odds ratio):1.95, 95% CI 1.11, 3.45], good attitude (AOR = 1.97, 95% CI = 1.04, 3.72), secondary school education level (AOR 2.91, CI 1.20, 7.01), diploma and above education level (AOR 4.33, 95% CI 1.41, 13.31), use of three compartment dish-washing system (AOR 2.47, CI 1.27, 4.80) and use of refrigerator (AOR 3.93, CI 1.79, 8.63) were factors statistically associated with good food handling practice. This study indicated that food handling practice was relatively poor. Work experience, good attitude, level of education, use of three compartment dishwashing systems and refrigerator were factors associated with food handling practice. Hence, structuring the kitchen with modern dish washing system and refrigerator would enhance good food handling practice.

**Electronic supplementary material:**

The online version of this article (10.1186/s13104-019-4047-0) contains supplementary material, which is available to authorized users.

## Introduction

Food can be contaminated from production up to consumption. Producers, shippers, processors, distributers and food handlers have the responsibility in ensuring the safety of food [1]. Particularly, in public food establishments, food handlers are the first responsible bodies to contaminate food by acting as either a biological or a physical carrier for many pathogenic organisms [2]. The degree of food contamination is highly determined by the degree of contact [2, 3].

Food contamination mainly occurs through poor food handling practice which results in numerous food borne diseases. These diseases are the major causes of morbidity and mortality. Globally, more than 50% of the total food poisoning cases were attributed to improper food handling procedure [4]. Around 600 million food borne illnesses and 420,000 deaths occur each year due to poor food handling practice [5]. Among ten people, one becomes ill from ingestion of contaminated food [5, 6]. In developing countries, approximately 10 to 20% of food-borne disease (FBD) outbreaks are due to food contamination [2]. South-east Asia and Africa constitutes the highest burden [7]. Around 700,000 people die due to FBD in sub-Saharan Africa [8]. In Ethiopia, though there is limited data, there were 280,458 out-patient cases in 2013 [9].

Hazardous pathogens and physical hazards like hand watch may causes life threatening health problems if they come in contact with food [2, 10–13]. Such Contaminants get access to contaminate food mainly due to food handler’s poor knowledge and negligence during handling activities. Moreover, low financial resources, inadequacy food safety law, in availability of food establishment guideline and standards, as well as poor monitoring and evaluation system of food establishments play an important role in food handling practice [1, 14].

In Ethiopia, the coordination activities particularly at lower levels of government bodies are so weak. There is no clearly defined responsibility to control, monitor and evaluate food handlers of food establishments [9]. Ethiopian Studies conducted in Diredawa and Arbaminch showed that prevalence of good food handling practice was 52.4% and 32.6% respectively [15, 16].

Therefore, it is crucial to conduct a repeated study aiming at assessing food handling practice and associated factors among food handlers in public food establishments in Debark town, North-west Ethiopia.

## Main text

### Methods

An institution based cross-sectional study was conducted from January 1 to 25, 2018 in Debark town, Northwest Ethiopia. The town is located 826 km Northwest of Addis Ababa, capital of Ethiopia. Within the town, there are 32 restaurants, 24 hotels, 49 cafes, 8 butcher houses and 3 juice houses which provide food for the catchment population and guests. All food handlers who were working in selected public food establishments in Debark town were the study population.

Sample size was estimated using single population proportion formula by considering food handling practice 52.4% [16], 5% margin of error at 95% confidence level and adding 10% non response rate the final sample size was 422. This study comprises selecting public food establishments like hotels, restaurants, cafes, butcher shops and juice houses. Proportional allocation was done and 170,189,166, 22 and 4 food handlers were recruited from hotels, restaurants, cafes, butcher and juice houses respectively. Finally, simple random sampling technique was used to select 422 food handlers.

Food handling practice was computed by using 21 questions and 29 observational check-lists totally 50 questions. Accordingly, participants who respond 70% and above considered as having good food handling practice whereas those who respond below 70% considered as having poor food handling practice [16, 17]. Knowledge towards food handling was assessed by 20 questions and participants who respond 70% and above considered as having good knowledge whereas below 70% were considered as having poor knowledge [16, 17]. Ten questions were used to assess attitude towards good food handling and participants who respond 80% and above considered as having good attitude whereas below 80% considered as having poor attitude [14].

Data was collected using structured interviewer administered questionnaire and observational checklist. It was adopted from World Health Organization (WHO) [3] and published studies with some modifications by adding socio-demographic variables, knowledge, attitude of food handler and sanitary condition of the food establishments. Pre-test was done on 5% food handlers found in a nearby town and necessary amendments were made. Close supervision was done during data collection. Completeness of the collected data was checked daily. Data were coded and entered into EPI info version 7 and then exported to SPSS version 20 statistical package for analysis. Descriptive statistics was carried out. Binary logistic regression model was employed and variables having p-value of < 0.2 were exported to multi-variable binary logistic regression model to identify confounders. Adjusted odds (AOR) with 95% CI and p value < 0.05 was used to show association. Variables having p-value ≤ 0.05 were taken as significantly associated with good food handling practice (Additional file 1).

### Results

#### Socio-demographic characteristics of food handlers

In this study a total of 416 food handlers were participated with a response rate of 416 (98.6%). Majority 379 (91.9%) of the participants were females. The median age of respondents was 21 years with inter-quartile range of ± 7. Of the total, 85.3% had taken primary education and above. Regarding monthly income, more than half 282 (67.8%) were earned 200–600 Ethiopian birr (Table 1).

**Table 1:** Socio-demographic characteristics of food handlers working in public food establishments in Debark town, North-west Ethiopia, 2018 (n = 416)

Variables	Category	Frequency (n)	Percent (%)
Sex	Female	379	91.9
	Male	37	8.9
Age in years	< 18	117	28.1
	18–21	106	25.5
	22–25	94	22.6
	> 25	99	23.8
Religion	Orthodox	394	94.7
	Muslim	22	5.3
Current marital status	Unmarried	326	78.4
	Married	90	21.6
Educational level	Informal	61	14.7
	Primary (1–8)	154	37
	Secondary (9–12)	156	37.5
	Diploma and above	45	10.8
Job responsibility	Cooker	207	49.8
	Waiter	174	41.8
	Washer	35	8.4
Monthly income	< 200 birr	27	6.5
	200–600 birr	282	67.8
	> 600	107	25.7
Work experience in different public food establishments	Yes	127	30.5
	No	289	69.5
Work experience (years)	< 1	215	51.7
	1–3	116	27.9
	> 3	85	20.4
Training	Yes	29	7
	No	387	93
License	Yes	28	6.7
	No	388	93.3
Supervision by government/owner/manager	Yes	199	47.8
	No	217	52.2
Feedback	Given	338	81.3
	Not given	78	18.8
Medical checkup	Yes	59	14.2
	No	357	85.8
Deworming	None	399	95.9
	Only once	13	3.1
	Twice a year	4	1
Working hour	< 8 h	93	22.4
	≥ 8 h	323	77.6

#### Knowledge and attitude of food handlers

Three hundred twenty-nine (79.1%) and 289 (69.5%) of respondents were found to have good knowledge and good attitude towards food handling practice respectively.

#### Institutional characteristics

All public food establishments had pipe water supply. About three-fourth 307 (73.8%) and majority 346 (83.2%) of respondents handled food in an institution having three compartment dishwashing system and functional toilet facility respectively. However, half 211 (50.7%) of respondents handled food in food establishments where there were no liquid waste storage (Table 2).

**Table 2:** Institutional characteristics of food handling practice among food handlers working in public food establishments in Debark town, North-west Ethiopia, 2018 (n = 416)

Variables	Category	Frequency (n)	Percent (%)
Liquid waste storage system	Yes	205	49.3
	No	211	50.7
Type of liquid waste storage system	Open space	179	43
	Sewage pit	140	33.7
	Septic tank	67	16.1
	Storm draining water	24	5.8
Insects or rodents breeding around establishment	Yes	112	26.9
	No	304	73.1
Functional kitchen	Yes	353	84.9
	No	63	15.1
Functional shower facility	Yes	159	38.2
	No	257	61.8
Clean utensils availability	Yes	363	87.3
	No	53	12.7
Shelf/cupboard availability	Yes	241	57.9
	No	175	42.1
Functional refrigerator in the kitchen	Yes	115	27.6
	No	301	72.4
Separate dressing room for food handler	Yes	97	23.3
	No	319	76.7

#### Food handling practice

In this study, the overall magnitude of good food handling practice was 40.1% (95% CI 35.10, 44.50).

#### Factors associated with food handling practice

Multi variable logistic regression output showed that work experience in different food establishments, good attitude, secondary school and diploma and above education levels, use of three compartment dishwashing system and refrigerator in the kitchen were factors associated with good food handling practice.

The odds of performing good handling practice among food handlers who had work experience in different public food establishments was two folds higher as compared to those food handlers who had no experience of work in such institutions (AOR = 1.95, 95% CI 1.11, 3.45).

Similarly, the odds of performing good handling practice among food handlers who had good attitude towards good food handling practice was twofolds higher as compared to their counterparts (AOR = 1.97, 95% CI 1.04, 3.72).

The result also showed that the odds of performing good food handling practice among food handlers who attended secondary school (grade 9–12) was three folds (AOR = 2.91, CI 1.20, 7.01) and those who had diploma and above was four and half folds (AOR = 4.33, CI 1.41, 13.31) higher as compared to those food handlers who were informally educated (illiterate and can read and write).

In addition, the likelihood of performing good food handling practice among food handlers who used three compartment dishwashing system was two and half folds (AOR = 2.47, CI 1.27, 4.80) higher than those food handlers who handled without using three compartment dishwashing system.

Furthermore, the odds of performing good food handling practice among food handlers who had functional refrigerator in their kitchen four folds (AOR = 3.93, CI 1.79, 8.63) higher as compared to those food handlers who had no functional refrigerator in their kitchen (Table 3).

**Table 3:** A bi-variable and multi-variable logistic regression analysis output of associated factors with food handling practice in Debark town, North-west Ethiopia, 2018 (n = 416)

Variable	Practice		COR (95% CI)	AOR (95% CI)
	Good	Poor		
Monthly income				
< 200	8 (29.6%)	19 (70.4%)	1	1
200–600	98 (34.8%)	184 (65.2%)	1.27 (0.53–2.99)	0.45 (0.15–1.35)
> 600	61 (57%)	46 (43%)	3.15 (1.27–7.83)	0.35 (0.10–1.25)
Work experience (year)				
< 1	72 (33.5%)	143 (66.5%)	1	1
1–3	54 (46.6%)	62 (53.4%)	1.73 (1.09–2.75)	1.27 (0.69–2.35)
> 3	41 (48.2%)	44 (51.8%)	1.85 (1.11–3.09)	1.57 (0.74–3.32)
Working hour (h)				
≤ 8	137 (32.3%)	186 (67.7%)	1	1
> 8	30 (42.4%)	63 (57.6%)	0.65 (0.40–1.05)	0.56 (0.30–1.05)
Work experience in different public food establishments				
Yes	70 (55.1%)	57 (44.9%)	2.43 (1.59–3.72)	1.95 (1.11–3.45)*
No	97 (33.6%)	192 (64.4%)	1	1
Training				
Yes	18 (62.1%)	11 (37.9%)	2.61 (1.20–5.69)	1.33 (0.35–4.98)
No	149 (38.5%)	238 (61.5%)	1	1
License				
Yes	18 (64.3%)	10 (35.7%)	2.89 (1.30–6.42)	0.92 (0.24–3.54)
No	149 (38.4%)	239 (61.6%)	1	1
Supervision				
Yes	98 (49.2%)	101 (50.8%)	2.01 (1.40–3.10)	1.30 (0.78–2.16)
No	69 (31.8%)	148 (68.2%)	1	1
Medical check-up				
Yes	36 (61.0%)	23 (39.0%)	2.70 (1.53–4.75)	0.96 (0.43–2.19)
No	131 (36.7%)	226 (33.3%)	1	1
Building ownership				
Owned	104 (49.1%)	108 (50.9%)	2.16 (1.44–3.22)	1.15 (0.65–2.04)
Rented	63 (30.9%)	141 (69.1%)	1	1
Attitude				
Good	132 (45.7%)	157 (54.3%)	2.21 (1.41–3.48)	1.97 (1.04–3.72)*
Poor	35 (27.6%)	92 (72.4%)	1	1
Knowledge				
Good	145 (44.1%)	184 (55.9%)	2.33 (1.37–3.96)	1.31 (0.64–2.65)
Poor	22 (25.3%)	65 (74.7%)	1	1
Level of education				
Informal	14 (23.0%)	47 (77.0%)	1	1
Primary	51 (33.1%)	103 (69.9%)	1.68 (0.84–3.30)	1.83 (0.76–4.40)
Secondary	82 (47.4%)	74 (52.6%)	3.03 (1.54–5.95)	2.91 (1.20–7.01)*
Diploma and above	28 (62.2%)	17 (37.8%)	5.53 (2.37–12.91)	4.33 (1.41–13.31)**
Three compartment dishwashing system				
Yes	149 (48.5%)	158 (51.5%)	4.77 (2.74–8.29)	2.47 (1.27–4.80)*
No	18 (16.5%)	91 (83.5%)	1	1
Liquid waste disposal				
Yes	102 (49.8%)	103 (50.2%)	2.22 (1.49–3.32)	1.29 (0.77–2.16)
No	65 (30.8%)	146 (69.2%)	1	1

### Discussion

Improper food handling practice is one of the major routes for food born disease transmission. An emphasis need to be given for food handling practice by the concerned bodies. Therefore, this study provides an insight on status of food handling practice on the area. This study revealed that good food handling practice was 40.1% (95% CI 35.10, 44.50). The result is consistent with the study conducted in Nigeria 36.50% [18].

However, the finding is lower than other studies conducted in Malaysia 59.30% [19], Jordan 89.43% [20]. The variation might come across due to study settings. The study in Malaysia was conducted in university campus where as in Jordan the study was conducted in hospital. In fact, these institutions assumed to have adequate resources and suitable setup for food handling practice as compared to this study. In addition to this, the education level of food handlers in Malaysia and Jordan might contribute to the variation. Proportion of food handlers who attended secondary school and above were 77% and 94% in Malaysia and Jordan studies respectively where as in this study only 48.3%. As education level progresses, food handlers would have improved knowledge and attitude towards good food handling practice [21].

The result of this study is also lower than studies conducted in Dangla 52.5% [2], Dire Dawa 52.4% [16]. The discrepancy might be due to that a study conducted in Dangla used mean as cut off point to determine the prevalence of food handling practice but in this study percentage was used. Sensitivity of food to hot environment in Diredawa might make food handlers curious while handling [16] because foods may become easily perishable.

However, the results of this study is higher than other studies conducted in Gondar 30.3% [14], Gamogofa 32.6% [15]. This might be due to difference in year of study and cut off points used. The study conducted in Gondar town got around 5 years long. Due to globalization access of information improved from time to time and food handlers can develop good knowledge and positive attitude towards food handling so that they could perform good handling practice relatively better [22]. In addition to this, the cut off points used to determine food handling practice were different (80–100% = good, 60–79% = fair and < 60% = poor) as compared to this study in which food handling practice was determined in two levels using the minimum cut off point (≥ 70% = good and < 70 = poor) [16, 17]. Obviously, the cut-off point variation entirely alters the results of the study. In Gamogofa interviewees were mostly males and having primary school and below 68.66% as compared to this study in which only 8.9% of males and 51.7% of primary and below education level of respondents involved. This is because females had more experience even in their day to day home activity than males and low education level of food handlers will have poor knowledge and attitude so that not liable to apply basic good food handling principles [16].

The odds of performing good handling practice among food handlers who had experience was 1.95 times higher as compared to those who hadn’t. The possible explanation to this could be because experience could help food handlers to acquire better knowledge and skills regarding food handling practice.

Similarly, the odds of performing good handling practice among food handlers who had good attitude towards good food handling practice was 1.97 times higher as compared to their counterparts. Those who have good attitude are assumed to have good knowledge which is the foundation of skill/practice. This is also evidenced by other studies Gondar [14] and Diredawa [16].

The odds of performing good food handling practice among food handlers who had diploma and above 4.33 times higher as compared to those having no formal education. The result is supported by studies conducted in Italy [23], Jordan [20], Ghana [24], Diredawa [16], Nigeria [25] and Addis Ababa [21].

The likelihood of performing good food handling practice among food handlers who used three-compartment dishwashing system was 2.47 higher than don’ts. Moreover, the odds of performing good food handling practice among food handlers who had a functional refrigerator 3.93 times higher as compared to those who had no. This might be explained by better institutional infrastructure support and enhances better food handling practice [26]. This is in line with a study conducted in Diredawa [16].

In this study, food handling practice was relatively poor. Work experience, good attitude, level of education, use of three compartment dishwashing systems and refrigerator were factors associated with food handling practice. Food establishments better to hire food handlers having experience and secondary education and above. In addition, it is better to structuring the kitchen with modern dishwashing system and refrigerator.

## Limitations

The study might be liable to social desirability and recall bias. In addition, the cross-sectional nature of the study might affect the cause and effect relationship. Moreover, parasitic and microbiological laboratory analyses were not considered in this study.

## Additional file

**Additional file 1.** Questionnaires.

## Abbreviations

AOR: adjusted odds ratio
CI: confidence interval
COR: crude odds ratio
FBDs: food borne diseases
IRB: Institutional Review Board
SPSS: statistical package for social sciences
WHO: World Health Organization
