# Supplementary material for: Food handling practice and associated factors among food handlers in public food establishments, Northwest Ethiopia
Source: BMC Res Notes. 2019 Jan 14;12:20. doi: 10.1186/s13104-019-4047-0 (PMC6332519; doi:10.1186/s13104-019-4047-0)
Supplement: Supplementary file 1 — Additional file 1. Questionnaires. [file 13104_2019_4047_MOESM1_ESM.docx]

**English version questionnaires**

University of Gondar College of Medicine and Health Science, Institute of Public Health Structured questionnaire on factors affecting food handling practice in public food establishments in Debark town, North West Ethiopia, 2018.

| S.no | | Part 1 ፡Socio Demographic factors | | | skip |
| --- | --- | --- | --- | --- | --- |
| SD101 | | In which type of public food establishment you are working? | | 1.Hotel  2.Restaurant  3.Cafe  4.Butcher shop  5.Juice house |  |
| SD102 | | How old are you? | | ----------years |  |
| SD103 | | What is your sex? | | 1.Female  2.Male |  |
| SD104 | | What is your religion? | | 1.Orthodox 3.Catholic  2.Muslim 4.Protestant  5. Others specify------- |  |
| SD105 | | What is your marital status | | 1.Married 3.Divorced  2.Single 4.widowed  5.separated |  |
| SD106 | | What is your educational level? | | 1.unable to read and write  2. able to read and write  3.primary level(1-4)  4. primary level(5-8)  5.secondary level(9-10)  6. secondary level(11-12)  7.college level and above |  |
| SD107 | | What is your work responsibility | | 1.cooker  2.waiter  3.washer |  |
| SD108 | | What is your monthly income in Ethiopian birr? | | ---------birr |  |
| SD109 | | How long have you been working? | | ---------years |  |
| SD110 | | Have you ever worked in different public food establishments? | | 1.Yes  2.No |  |
| SD111 | | Have you ever been given food safety training? | | 1.Yes  2.No |  |
| SD112 | | If “YES” to Que.no.111, who trained you? | | 1 Government bodies  2.Owner/manager  3. other specify---------- |  |
| SD113 | | Do you have a license? | | 1.Yes  2.No |  |
| SD114 | | Have you ever been supervised? | | 1.Yes  2.No (skip to 117) |  |
| SD115 | | If “Yes” to Que.no.114,who had supervised you? | | 1.Government bodies  2.owner/manager  3. other specify---------- |  |
| SD116 | | If “Yes” to Que.no.114, how often did they supervise you in the last year? | | 1.Any time  2.every month  3.every three month  4.twice a year  5.once a year |  |
| SD117 | | Have you been given feedback from the customer? | | 1.Yes  2.No |  |
| SD118 | | Did you do a regular medical check-up in the last year? | | 1. Yes  2.No ( skip to 120) |  |
| SD119 | | If<YES> to que.no.118, how many times did you do per a year? | | 1.monthly  2.three times per a year  3.twice per a year  4.once per a year |  |
| SD120 | | How many times have you been de-wormed within a year? | | 1. every six month  2. only once  3. not at all |  |
| SD121 | | For how long do you work per a day? | | 1.for six hour  2.four to eight hour  3.for twelve hour  4.for >twelve hour |  |
| SD122 | | For whom the establishment building belongs? | | 1.Owned  2.Rented |  |
| **Part2: Food handler’s knowledge question**  **Part2.1Transmission of foodborne diseases** | | | | |  |
| K201 | Fresh/ raw meat may have harmful microbes on the surface | | 1.Yes  2.No  3.I don’t know | | skip |
| K202 | | Healthy/ asymptomatic food handler can cause illness by carrying germs to food | 1.Yes  2.No  3.I don’t know | |  |
| K203 | | Lettuce and other raw vegetables might have harmful microbes | 1.Yes  2.No  3.I don’t know | |  |
| K204 | | Well cooked foods do not have microbes | 1.Yes  2.No  3.I don’t know | |  |
| **Part2.2 Personal health and hygiene** | | | | |  |
| K205 | | You can prepare food with a wound on the hand if the wound is covered with a bandage | 1.Yes  2.No  3.I don’t know | |  |
| K206 | | It is enough to wash the Hands with water alone after handling raw meat | 1.Yes  2.No  3.I don’t know | |  |
| K207 | | After using the toilet, we should always wash hands with soap and water | 1.Yes  2.No  3.I don’t know | |  |
| K208 | | Habit of finger nose is dangerous | 1.Yes  2.No  3.I don’t know | |  |
| K209 | | When wearing gloves, you can handle cooked foods after handling raw meat | 1.Yes  2.No  3.I don’t know | |  |
| K210 | | Wearing clean uniform and cap while cooking or serving is necessary | 1.Yes  2.No  3.I don’t know | |  |
| K211 | | Hands should be properly washed after sneezing or blowing your nose | 1.Yes  2.No  3.I don’t know | |  |
| **Part2.3 Contamination/cross contamination** | | | | |  |
| K212 | | Food-borne disease can result from storing raw meat and cooked foods in the same refrigerator | 1.Yes  2.No  3.I don’t know | |  |
| K213 | | Washing fruits/ vegetables before preparing/eating is not important | 1.Yes  2.No  3.I don’t know | |  |
| K214 | | Ready to eat foods (e.g., vegetables) can be prepared on the same cutting board that was used to prepare meat | 1.Yes  2.No  3.I don’t know | |  |
| K215 | | Cutting boards, meat slicers and knives should be sanitized after each use | 1.Yes  2.No  3.I don’t know | |  |
| **Part2.4Temperature control** | | | | |  |
| K216 | | Foods like egg, meat, milk that need to be kept hot should cooked upto bubble emission or 60° C and above | 1.Yes  2.No  3.I don’t know | |  |
| K217 | | Leftovers should be reheated to a minimum temperature of 70°C or more than the first cooking time | 1.Yes  2.No  3.I don’t know | |  |
| K218 | | Foods should be slowly cooled at room temperature before storage in the refrigerator | 1.Yes  2.No  3.I don’t know | |  |
| K209 | | Refrigeration kills all the bacteria that might cause food-borne illnesses | 1.Yes  2.No  3.I don’t know | |  |
| K220 | | Foods like egg, meat, milk which needs to be kept hot should be cooked for more than 15 minutes | 1.Yes  2.No  3.I don’t know | |  |

| ***Part3: Food handlers attitudinal questions*** | | | | ***skip*** |
| --- | --- | --- | --- | --- |
| *A301* | Food borne disease causing microorganisms found every where | 4Strongly Agree  3Agree  2 Neutral  1Disagree  0Strongly Disagree | |  |
| *A302* | Temperature Controls are an effective method of reducing the number of cases of food poisoning | 4 Strongly Agree  3 Agree  2 Neutral  1 Disagree  0 Strongly Disagree | |  |
| A303 | All food handlers should have a food safety training qualification | 4 Strongly Agree  3 Agree  2 Neutral  1 Disagree  0 Strongly Disagree | |  |
| A304 | Expired food never serve even they are not changed in the smell and taste | 4 Strongly Agree  3 Agree  2 Neutral  1 Disagree  0 Strongly Disagree | |  |
| A305 | Food handlers who have discharge from nose,ear, eye and skin lesion should be away from work for atleast two days and see a doctor | 4 Strongly Agree  3 Agree  2 Neutral  1 Disagree  0 Strongly Disagree | |  |
| A306 | Safe food handling to avoid contamination and disease is my part job responsibility | 4 Strongly Agree  3 Agree  2 Neutral  1 Disagree  0 Strongly Disagree | |  |
| A307 | Hand washing before handling food reduces the risk of contamination | 4 Strongly Agree  3 Agree  2 Neutral  1 Disagree  0 Strongly Disagree | |  |
| A308 | Food can be in contaminated at any occasion from production up to consumption | 4 Strongly Agree  3 Agree  2 Neutral  1 Disagree  0 Strongly Disagree | |  |
| A309 | Unavailability of food handling guideline can affect food safety | 4 Strongly Agree  3 Agree  2 Neutral  1 Disagree  0 Strongly Disagree | |  |
| A310 | Lack of supervisor commitment affect safe food handling | 4 Strongly Agree  3 Agree  2 Neutral  1 Disagree  0 Strongly Disagree | |  |
|  | | | | |
| **Part4: personal practical questions** | | | |  |
| PQ401 | Do you cut your finger nail weekly? | | 1.Always  2. Sometimes  3.Never |  |
| PQ402 | Do you prepare food having discharge from nose/ear/eye/skin? | | 1.Always  2. Sometimes  3.Never |  |
| PQ403 | Do you prepare food tie with bandage while a cut? | | 1.Always  2. Sometimes  3.Never |  |
| PQ404 | Do you wear hand jewelry like watch, bras let and ring other than wedding ring? | | 1.Always  2. Sometimes  3.Never |  |
| PQ405 | Do you put nail paint on your nail while preparing food? | | 1.Always  2. Sometimes  3.Never |  |
| PQ406 | Do you wear hair cover while preparing food? | | 1.Always  2. Sometimes  3.Never |  |
| PQ407 | Do you wear gown while preparing food? | | 1.Always  2. Sometimes  3.Never |  |
| PQ408 | Do you wash your hands before starting work | | 1.Always  2. Sometimes  3.Never |  |
| PQ409 | Do you wash your hands before handling ready-to-eat foods | | 1.Always  2. Sometimes  3.Never |  |
| PQ410 | Do you wash your hands after preparing raw foods | | 1.Always  2. Sometimes  3.Never |  |
| PQ411 | Do you wash your hands after touching body parts/other things | | 1.Always  2. Sometimes  3.Never |  |
| PQ412 | Do you wash your hands after using the toilet | | 1.Always  2. Sometimes  3.Never |  |
| PQ413 | When you wash your hands, do you use soap with water? | | 1.Always  2. Sometimes  3.Never |  |
| PQ414 | Do you use hot water to wash your hands | | 1.Always  2. Sometimes  3.Never |  |
| PQ415 | Do you use hot water to wash food contact utensils | | 1.Always  2. Sometimes  3.Never |  |
| PQ416 | Do you clean the working surfaces after each task? | | 1.Always  2. Sometimes  3.Never |  |
| PQ417 | Do you store raw and cooked food separately? | | 1.Always  2. Sometimes  3.Never |  |
| PQ418 | Do you cover cooked food? | | 1.Always  2. Sometimes  3.Never |  |
| PQ419 | Do you check the expiry dates of all products? | | 1.Always  2. Sometimes  3.Never |  |
| PQ420 | Do you use a handkerchief or rag when suffering from a cold? | | 1.Always  2. Sometimes  3.Never |  |
| PQ421 | Did food handler have the following symptoms in the last two weeks(diarrhea, abdominal cramp, vomiting, fever, jaundice, pusy-discharge of any site) | | 1.Always  2. Sometimes  3.Never |  |

| **Part5: Observational check-list**  **OC5.1 Food handler’s personal condition** | | | | | **skip** |
| --- | --- | --- | --- | --- | --- |
| OC501 | | Is food handler’s finger nail trimmed and cleaned? | | 1.Yes  2. No |  |
| OC502 | | Are there dirty particles observed under fingernail | | 1.Yes  2. No |  |
| OC503 | | Do you observe discharge from nose,ear,eye and cough from handlers during visit? | | 1.Yes  2. No |  |
| OC504 | | Any visible skin rash, boils, cut, wound from food handler at time of visit? | | 1.Yes  2. No |  |
| OC505 | | Do you observe wearing hand jewelry/ring other than wedding ring during visit? | | 1.Yes  2. No |  |
| OC506 | | Do you observe finger paintingduring visit? | | 1.Yes  2. No |  |
| OC507 | | Does food handler put on clean hair cover during visit? | | 1.Yes  2. No |  |
| OC508 | | Does food handler wear clean gown during visit? | | 1.Yes  2. No |  |
| OC509 | | Does the food handler wash his/her hands with soap and water before starting work | | 1.Yes  2. No |  |
| OC510 | | Does the food handler wash his/her hands with soap and water before handling ready-to-eat foods | | 1.Yes  2. No |  |
| OC511 | | Does the food handler wash his/her hands with soap and water after preparing raw foods | | 1.Yes  2. No |  |
| OC512 | | Does the food handler wash his/her hands with soap and water after touching body parts/other things | | 1.Yes  2. No |  |
| OC513 | | Does the food handler wash his/her hands with soap and water after using toilet | | 1.Yes  2. No |  |
| OC514 | | Does the food handler use hot water for washing hands during visit? | | 1.Yes  2. No |  |
| OC515 | | Does the food handler follow proper hand-washing procedure? | | 1.Yes  2. No |  |
| OC516 | | Does the food handler use hot water for washing dishes during visit? | | 1.Yes  2. No |  |
| OC517 | | Does the food handler use soap/detergents for washing dishes during visit? | | 1.Yes  2. No |  |
| OC518 | | Does the food handler clean and sanitize the work surface after each task at the time of observation? | | 1.Yes  2. No |  |
| OC519 | | Does the food handler keeps ready to eat foods in a clean container and covered properly? | | 1.Yes  2. No |  |
| OC520 | | Does the food handler use hand glove, ladles, spoons, tongs, scoops while handling ready to eat foods? | | 1.Yes  2. No |  |
| OC521 | | Does the food handler place food utensils in a well arranged manner in a self/cupboard? | | 1.Yes  2. No |  |
| OC522 | | Does the food handler storeraw food item in area separate from cooked food? | | 1.Yes  2. No |  |
| OC523 | | Does the food handler store perishable ready to eat food in refrigerator? | | 1.Yes  2. No |  |
| OC524 | | Do you use separate utensils when preparing raw and cooked foods? | | 1.Yes  2. No |  |
| OC525 | | Does the food handler use kitchen towels to dry dishes after washed? | | 1.Yes  2. No |  |
| OC526 | | Does the food handler have separate shoes in the food establishment? | | 1.Yes  2. No |  |
| OC527 | | Does food handler collect and discard left-overs | | 1.Yes  2. No |  |
| OC528 | | Do you observe food ruminants on the table/floor during visit? | | 1.Yes  2. No |  |
| OC529 | | Does the food handler frequently clean the dining table with clean cloth after each use? | | 1.Yes  2. No |  |
| **OC5.2 Sanitary condition of public food establishments (Observation)** | | | | |  |
| OC530 | Is your source of water come from pipe? | | 1.Yes  2. No | |  |
| OC531 | If your answer for question 530 is “yes”, the type of pipe is? | | 1.Private pipe  2.Pipe shared  3.Pipe from neighbor | |  |
| OC532 | Are there three compartment dishwashing systems? | | 1.Yes  2.No | |  |
| OC533 | Is there functional toilet facility available? | | 1.Yes  2.No  (If no skip to 536 ) | |  |
| OC534 | If your answer for question 533is “yes”, is there a separate toilet for food handlers? | | 1.Yes  2.No | |  |
| OC535 | If your answer for question 533 is “yes”, is there hand washing basin near the toilet? | | 1.Yes  2.No | |  |
| OC536 | Is there a container for solid waste storage? | | 1.Yes  2.No  (If no skip to 538 ) | |  |
| OC537 | If the answer for question 536 is “yes”, what type of solid waste storage container is available? | | 1.Dust bin  2.Barrel  3.Sack  4.Others, specify | |  |
| OC538 | Is there liquid waste disposal? | | 1.Yes  2.No  (If no skip to 540 ) | |  |
| OC539 | If the answer for Que.538 is “yes”,where does waste water from hand washing and dishwashing facilities disposed-off? | | 1.Open space  2.Septic tank  3.Latrine  4.Storm-water drainage  5.Seepage pit  6.Other | |  |
| OC540 | Is there any insects breeding around the food establishment center? | | 1.Yes  2.No | |  |
| OC541 | Does the food establishment have a kitchen? | | 1.Yes  2.No(If no skip to 544 ) | |  |
| OC542 | If “yes” to Que. 541, is the kitchen wall and ceiling free of dust, spider web and smoke particles | | 1.Yes  2.No | |  |
| OC543 | If “yes” to Que. 541, is there hand washing facility with detergent with in the kitchen? | | 1.Yes  2.No | |  |
| OC544 | Is there functional shower facility available? | | 1.Yes  2.No | |  |
| OC545 | Are there clean/neat utensils in the kitchen? | | 1.Yes  2.No | |  |
| OC546 | Is there cooking control thermometer in the kitchen? | | 1.Yes  2.No | |  |
| OC547 | Is there a shelf/cupboard in the kitchen | | 1.Yes  2.No | |  |
| OC548 | Is there functional refrigerator in the kitchen? | | 1.Yes  2.No | |  |
| OC549 | Is there separate dressing room for food handlers? | | 1.Yes  2.No | |  |
